# Review of Prognostic Significance of Quantitative BPE Measurements

**DOI:** 10.3390/diagnostics16030495

**Published:** 2026-02-06

**Authors:** Jeremy Weiss, Emily Hunt, Yihui Zhu, Tim Q. Duong, Takouhie Maldjian

**Affiliations:** 1 Department of Radiology, Montefiore Medical Center, Albert Einstein College of Medicine, New York, NY 10467, USA; jeremy.weiss@einsteinmed.edu (J.W.); yihui.zhu@einsteinmed.edu (Y.Z.); tim.duong@einsteinmed.edu (T.Q.D.); 2 Department of Radiology, School of Medicine, Stanford University, Palo Alto, CA 94305, USA

**Keywords:** prognosis, biomarkers, radiology, breast neoplasms, female

## Abstract

**Background/Objectives**: Background parenchymal enhancement (BPE) on breast magnetic resonance imaging reflects hormonal and vascular activity of fibroglandular tissue and is studied as a prognostic marker for breast cancer. This paper serves as a review that evaluates quantitative methods for BPE measurements for predicting treatment outcomes. **Methods**: PubMed was searched for papers on evaluating BPE with outcomes to compare, such as pathologic complete response, recurrence-free survival, disease-free survival, and overall survival, from 2015 to 2025. In total, eleven papers using quantitative methods to measure BPE were selected. **Results**: Quantitative results showed that BPE reduction during neoadjuvant chemotherapy and high pre-treatment/baseline BPE are linked to improved treatment response and reduced risk of recurrence. **Conclusions**: Quantitative assessment methods yield objective and reproducible prognostic information. Incorporating quantitative BPE measurements alongside tumor-focused imaging features may further improve predictive accuracy in clinical settings.

## 1. Introduction

Breast cancer is the most common type of cancer in women. Thus, it is important that accurate and accessible tools are available for assessing neoplasm aggressiveness to then individualize treatment. Traditionally, clinicopathologic parameters such as TNM classification, tumor histology, and receptor types have been used to evaluate breast cancer behavior, appropriate treatment, prognosis, and recurrence. In recent times, imaging biomarkers have been increasingly of interest for prognosis [1,2], risk stratification [3], treatment planning [4], evaluation of treatment response [5,6], recurrence [7], and distant metastasis [8] since they are non-invasive and widely available.

Background parenchymal enhancement (BPE) is an imaging feature seen on contrast-enhanced breast magnetic resonance imaging (MRI) that has been assessed in the literature as a potential biomarker for breast cancer. Specifically, BPE is a measure of the degree of contrast uptake in fibroglandular tissue (FGT), reflecting vascularity, and is influenced by age, breast density, and hormones. One recent systematic review suggested that a decrease in BPE during and after neoadjuvant chemotherapy (NAC) was associated with pathologic complete response (pCR), but the role of BPE as a prognostic factor was less clear [9]. A separate review also reported a decrease in BPE in women undergoing NAC [10].

In clinical radiology practice, BPE is typically a subjective imaging feature of contrast-enhanced breast MRI interpreted by the radiologist as minimal, mild, moderate, and marked using the breast imaging-reporting assessment and data system (BI-RADS). While the BI-RADS classification is useful for clinical practice, qualitative interpretations are prone to inter- and intra-observer variability, which may affect the predictive and prognostic strength of BPE as an imaging biomarker [11].

Quantitative measures have been used to better objectively evaluate BPE, although employment across institutions and systems has not been standardized. Quantitative methods of measuring BPE include semi-automated volumetric approaches to fully automated machine learning algorithms. Quantitative BPE has the potential to limit subjectivity and variability and improve reproducibility and consistency, which can not only better imaging precision but also further research in BPE as a biomarker [11]. In fact, one retrospective case–control study that investigated the prediction of breast cancer risk using a semi-automated segmentation algorithm out-performed the radiologist-assigned BPE value [12].

Prior review articles have explored the relationship between BPE and breast cancer characteristics and outcome [9,10,13]. However, given ongoing debate regarding the role of BPE as a predictive and prognostic biomarker, we propose that some of the inconsistent findings in the literature may stem from the use of subjective, non-quantitative BPE evaluations. To address this, our review focuses exclusively on studies employing quantitative BPE measurements to ensure methodological consistency and improved objectivity. In this review, we aim to critically appraise the previous literature on quantitative measures of BPE and their prognostic significance, specifically looking at the outcomes recurrence-free survival (RFS), disease-free survival (DFS), overall survival (OS), and pCR in breast cancer patients.

## 2. Materials and Methods

This review did not require institutional review board approval. The literature review was performed on PubMed from 2015 to 2025. Using specified keywords, 113 total articles were identified, 59 of which were duplicates. In addition, one paper, by van der Valen et al. [14], was also selected from the reference section of a 2021 review article by Rella et al. [9] due to its relevance despite not being identified in the initial article search, to make a total of 114 articles, with 53 articles remaining once 59 duplicate articles were removed.

Three independent reviewers screened all identified titles and abstracts for eligibility. Full-text articles were reviewed when eligibility was unclear. Inclusion criteria were studies that evaluated BPE quantitatively in invasive breast cancer patients and reported an outcome measure such as pCR, OS, DFS, or RFS. Exclusion criteria included review articles, studies not measuring BPE quantitatively, studies without the target clinical outcome(s) studied, research published outside the time range, and duplicate studies.

Ten search terms were used to identify studies for this review, five used by one investigator and five used by a different investigator. The first search term was “(“background parenchymal enhancement” OR “BPE”) AND “breast cancer” AND “pathologic complete response”,” which yielded 13 articles, some of which were not included due to systematic review format, non-independent analysis of BPE, qualitative measures of BPE, and utilization of parenchymal kinetics instead of BPE. A similar second search term “(background parenchymal enhancement [title]) AND (breast cancer) AND (pathologic complete response),” yielded 9 articles, all of which were duplicates except one.

The third keyword was “(“background parenchymal enhancement” OR “BPE”) AND “breast cancer” AND “overall survival”,” which yielded 4 papers, 3 of which were excluded due to their qualitative measure of BPE. A similar fourth search term was used, “(background parenchymal enhancement [title]) and (breast cancer) and (overall survival),” which yielded 10 papers, many of which were duplicates or included investigation into qualitative BPE measurements.

The fifth key term, “(“background parenchymal enhancement” OR “BPE”) AND “breast cancer” AND “recurrence free survival”,” yielded 7 articles, which had duplicates and papers to undergo further review. The sixth search term was similarly “(background parenchymal enhancement [title]) and (breast cancer) and (recurrence free survival),” which revealed all repeated papers.

The seventh search term, “(“background parenchymal enhancement” OR “BPE”) AND “breast cancer” AND “disease free survival”,” provided 11 papers, all of which were duplicates or qualitative BPE studies. The eighth search term was “(background parenchymal enhancement [title] AND (breast cancer) AND (disease free survival),” which yielded 7 duplicate articles.

The ninth search term, “(“background parenchymal enhancement” OR “BPE”) AND “breast cancer” AND “prognosis”,” yielded 31 papers, with several duplicates and articles lacking relevance or pertinent methodology. The tenth and final search term, “(background parenchymal enhancement [title]) and (breast cancer) and (prognosis),” resulted in 21 articles, all of which were duplicates or lacked relevance.

**Figure 1:**
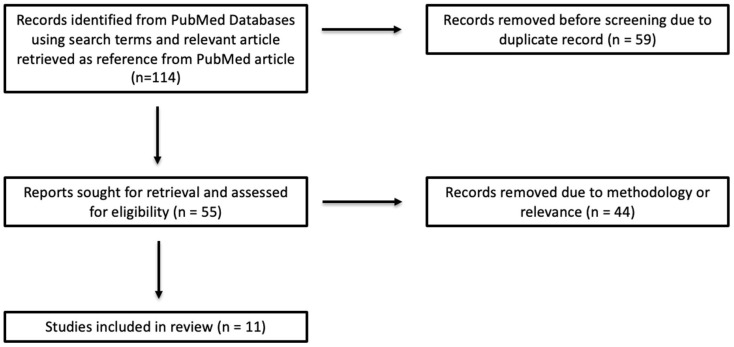
Article selection flowchart.

Data extraction was performed using a standardized template, which included author, year, study population, MRI acquisition protocol, BPE assessment method (quantitative), prognostic outcomes studied, and key findings. Ten articles were included in the review based on our inclusion and exclusion criteria, which is shown in our article selection flowchart (Figure 1).

## 3. Results/Discussion

Most qualitative BPE studies find that visually graded BI-RADS BPE, whether at baseline or as a change over time, does not independently predict survival (RFS, DFS, or OS) once standard clinical covariates are included [15,16,17]. Overall, when assessed qualitatively, BI-RADS BPE is a plausible indicator of parenchymal activity and treatment-related change but is not a reliable independent prognostic biomarker in unselected populations by itself. The clearest exception is the chemo-treated triple-negative breast cancer (TNBC) subgroup, in which higher contralateral BPE associates with improved survival endpoints [18]. Outside this setting, qualitative BPE remains non-prognostic. These patterns all together, with the variability inherent to qualitative reads, support a shift toward quantitative/semi-automated methods and integration with tumor-centric features to stabilize the signal and improve outcome modeling [15,16,17]. Therefore, we will focus our review on quantitative BPE methods relating to prognosis (Table 1).

Quantitative approaches to BPE consistently show that during NAC, the magnitude/direction of change carries much more information than any single baseline measurement. In particular, larger reductions in quantitative BPE over the course of treatment tend to track with better therapeutic response. This association is noted in some studies in HR-positive groups, where endocrine-responsive background tissue is common [14,19,20]. Other investigators found reduction in BPE associated with pCR in HR-negative patients, particularly early on during NAC [21,22,23]. Combining BPE with other features, such as sphericity, longest diameter, and functional tumor volume (FTV), has the potential to yield an even higher area under the receiver operating characteristic curve (AUC) [24].

Van der Velden et al. evaluated three-phase post-contrast pre-treatment MRI exams of 322 patients with ER-positive/HER2-negative invasive ductal breast cancer and found that high BPE on pre-treatment MRI in the disease-free contralateral breast is an independent biomarker for disease-free invasive cancer survival and overall survival in this cohort [14]. This study used automatic segmentation. Their BPE calculation was unique in that it was derived by calculating enhancement of the late phase (last of three post-contrast phases) at each voxel location as the relative increase in signal intensity between the first post-contrast scan and the third post-contrast scan, sorting lowest to highest of these late enhancement values, and evaluating the top 10% (values exceeding the 90th percentile), then calculating the mean of these top 10%. This highlights the broader relevance of parenchymal enhancement beyond treatment response prediction and illustrates how a standardized, quantitative approach and methodology can improve reproducibility and strengthens the clinical utility of this metric.

However, there is also asymmetry between pre- and post-treatment prognostic values. Pre-treatment quantitative BPE overall is not predictive of outcome in mixed cohorts. This reinforces the limited use of BPE for long-term risk stratification. In contrast, persistently high BPE after therapy has been linked to higher recurrence risk [25]. Moliere et al. evaluated 102 patients with biopsy-proven invasive breast cancer for post NAC response and demonstrated that quantitative BPE post-NAC, but not pre-NAC BPE, significantly predicted recurrence and correlated with DFS, independently of pCR [25]. Pathological complete response did not reach statistical significance, which was felt to be related to the small number of events during the follow-up period. Quantitative post-NAC BPE was significantly lower relative to pre-NAC BPE. Their quantitative method was more sophisticated and refined than the other studies, as they utilized a percent threshold where anything below that threshold was not included as BPE. They calculated VBPE as the total volume of the enhancing voxels over the fibroglandular region that had an enhancement ratio of greater than or equal to 20% (relative difference of 20% or greater). Their findings suggest that parenchyma following NAC carries prognostic information that is most likely not captured by just baseline values.

Timing of BPE measurement relative to therapy can significantly change performance, with several studies finding that early to mid-treatment timepoints often yield the most useful signal for response prediction [21,22,23,24]. Overall, longitudinal BPE can be a treatment response signal and potential prognostic marker post-NAC. Supporting these findings, the study by You et al. showed a continual reduction in BPE regardless of menopausal status, as well as a reduction in tumor size, throughout NAC treatment, with the reduction in BPE after second NAC demonstrating the highest AUC (0.726) for predicting pCR, especially in HR-negative patients [21]. You et al. examined change in BPE from pre-treatment BPE and second, fourth, and sixth NAC timepoints. These investigators utilized an automated three-step segmentation pipeline, demonstrating how standardizing BPE quantification can improve reproducibility and treatment response assessments.

Chen et al. examined change in BPE between baseline and two follow-up MRI exams and its relationship to pCR [22]. They found that pre-treatment BPE was higher in the pCR group, which on sub-group analysis was only seen in patients under age 55. They found that the change in BPE was significantly decreased on first follow-up MRI in the pCR group, which on subgroup analysis was only seen in the patients under age 55 and on receptor type stratification was only seen in the ER-negative cohort. In their study, the pCR rate was higher in ER-negative than ER-positive patients and higher in HER2-postive than HER2-negative patients, as would be expected. They also used a semi-automated segmentation method, further demonstrating how standardization of quantitative methodology can yield meaningful links between early BPE and NAC response.

Segmentation methods also matter. Nguyen et al. observed timepoint-specific differences in predictive performance of BPE with AUC that was optimized with the automated half-stack segmentation protocol versus automated full segmentation and versus automated segmentation of the central five slices [23]. Full-stack segmentation consisted of all axial slices containing FGT voxels, half-stack consisted of the central 50% of the latter, and the center five consisted of the central five slices. While %ΔBPE_02_ (later interval) showed potential predictive value in HR+/HER2− patients, the strongest associations appeared at earlier timepoints in HR−/HER2+ tumors treated with taxane-based NAC, suggesting that the timing at which BPE change is measured may significantly influence its use as an imaging biomarker. Statistically significant and highest AUC (0.87) for predicting pCR from change in BPE was noted in HR−/HER2+ at an early timepoint (T1) with half-stack segmentation, showing that segmentation methods matter. They suggest that BPE may serve as a good imaging biomarker in this cohort for early detection of pCR during NAC. A major limitation of their results is the small size of their HR−/HER2+ cohort (27 patients), of which 22 attained pCR.

Onishi et al. used a fully automated segmentation method, derived a voxel-by-voxel percent enhancement map, and averaged the percent enhancement of all voxels in the masked volume to determine BPE of the unaffected breast [20]. Pre-treatment (T0), early treatment (T1), inter-regimen (T2), and pre-surgical (T3) timepoints of BPE were evaluated. This study showed that insufficient suppression of background parenchymal enhancement (BPE) is linked to a poorer response to NAC in hormone receptor-positive patients, both after 12 weeks of treatment (inter-regimen point, T2) and at the pre-surgery timepoint (T3). Notably, the association observed at T2 suggests that early identification of patients with persistent BPE could help predict suboptimal response, enabling timely, personalized adjustments to their treatment plan.

Arasu et al. utilized manual whole breast segmentation, followed by deriving a mask classifying FGT and using fuzzy c-means clustering to remove non-breast elements [19]. Per voxel basis calculation of BPE was then performed with an average value of all voxels used to determine a final BPE estimate. They prospectively studied BPE at pre-treatment (T0), early treatment (T1), inter-regimen (T2), and pre-surgery (T3) in HR+/HER2− and HR−/HER2− patients. They studied 45 HR−/HER2− patients and 43 HR+/HER2− patients. They found that the change in BPE from baseline to pre-surgery was statistically significant in their cohort of 43 women with HR+/HER2− breast cancer undergoing taxane- and anthracycline-based regimens, with the highest AUC of 0.77 for predicting pCR for change in BPE between pre-surgery and pre-treatment/baseline timepoints. BPE of the contralateral unaffected breast demonstrated similar diagnostic accuracy compared to FTV for HR+/HER2− patients under univariate analysis. While BPE demonstrated potential to be an independent marker of response, their study found limited additive effect of BPE to FTV in predicting pCR, possibly related to the small sample size. Their findings demonstrate that the reaction of normal FGT to neoadjuvant therapy as reflected by BPE may serve as a biomarker of treatment response. The heightened sensitivity of changes in BPE for predicting pCR in HR+ tumors aligns with the known influence of estrogen on BPE. The progressive increase in both the magnitude and predictive strength of BPE at later timepoints in HR+ cancers suggests a consistent trend, making it less likely that these findings are due to random variation.

Li et al. utilized a fully automated segmentation method and demonstrated the following AUC for BPE in predicting pCR for various receptor types: 0.69 combined group of all receptor types, 0.66 for HR+/HER2−, 0.76 for HR+/HER2+, 0.75 for HR−/HER2+, and 0.62 for HR−/HER2− [24]. When combining all four features they examined (functional tumor volume, sphericity, longest diameter, and BPE), the AUC for the same receptor groups, respectively, were 0.81, 0.83, 0.88, 0.83, and 0.82. One hundred repeated five-fold cross validation was applied to ensure classification accuracy. Their study demonstrated that multifeature analysis was superior to any single feature in predicting pCR, which illustrates that the addition of BPE to prediction models enhances accuracy. Their study included 384 patients (162 HR+/HER2−, 60 HR+/HER2+, 30 HR−/HER2+, and 132 HR−/HER2−). The larger population size of their study compared to the study by Arasu et al. may explain why they were able to show that multifeature analysis that includes BPE is superior to univariable analysis. Across multiple analyses, quantitative BPE alone adds little value beyond tumor morphology, enhancement kinetics, and volumetric response measures. But when BPE is combined with FTV and biologic subtype, discrimination for pCR improves significantly. This indicates that background and tumor signals are complementary [19,24]. Thus, this pattern adds support for a combined modeling that uses background tissue change with other features, like volume and subtype, in NAC.

The only studies that failed to demonstrate correlation of change in BPE with pCR or RFS/DFS was conducted by Rella et al. and Shin et al., respectively [26,27]. The study by Rella et al. relied on initial manual segmentation of the fibroglandular tissue. Rella et al. also evaluated OS and DFS in a later study (2022) where they reported no significant associations between baseline BPE, final BPE, and change in BPE to outcomes [28]. Shin et al. performed quantitative BPE using three manual regions of interest (ROIs) placed for each study with average values for BPE calculated, in addition to a quantitative BPE assessment using fully automated segmentation of the FGT (to eliminate subjectivity) with later enhancement calculated for each voxel of FGT in the contralateral breast, taking the mean of the top 10 percent for analysis in ER+/HER2− node negative breast cancer patients, which showed no association with RFS or DFS [27]. They also used qualitative BPE assessment from two radiologists independently, also showing no association with RFS or DFS [27].

Overall, these findings highlight that quantitative BPE can behave differently across subtypes and treatment contexts, while also underscoring the sensitivity of BPE metrics to methodological factors such as study design and cohort characteristics. While full-breast, multi-timepoint analyses are ideal in theory, protocols that prioritize a standardized, reproducible automated segmentation methodology and a prespecified early or mid-NAC are most likely the best balance between feasibility and accuracy. Accordingly, contralateral quantitative BPE may be better regarded as a contextual imaging feature, with associations to prognostic variables that are sensitive to methodological factors, and that may enhance risk stratification within select subgroups rather than serve as a universal prognostic biomarker.

The literature reflects a clear shift in BPE quantification from single timepoint assessments to longitudinal approaches during NAC, giving importance to changes in BPE over time. Although pre- and post-treatment BPE comparisons have demonstrated utility as functional biomarkers of tumor response, they are limited in scope and fail to relay the tumor and microenvironmental changes that occur throughout NAC. Furthermore, analysis based on pre-treatment and post-treatment BPE values alone precludes the ability to assess early response to NAC, limiting opportunities for timely treatment adaptation. To address these limitations, contemporary studies increasingly employ longitudinal BPE analysis across multiple NAC timepoints, with promising results. This approach allows monitoring of temporal BPE dynamics, enabling earlier and more accurate prediction of treatment response and supporting more personalized treatment strategies.

The literature increasingly supports the adoption of fully automated approaches for quantifying BPE across multiple timepoints, as these methods demonstrate greater reliability than qualitative assessments. The results from the study by Rella et al. [26] illustrate how the limitations in accuracy and reproducibility associated with manual or semi-automated preliminary segmentation of the ROI can lead to results that diverge from the multitude of studies that employ automated methods. Accordingly, fully automated segmentation strategies are recommended to improve measurement robustness and consistency. A limitation to this strategy of purely automated segmentation of FGT lies in the lack of a standardized segmentation framework, which continues to introduce and propagate variability and potential error. These challenges underscore the need to transition toward deep learning-based methodologies. Such approaches offer the potential for fully automated, reproducible, and more precise segmentation, thereby improving BPE quantification and mitigating limitations inherent to existing methods. Consistent with this, comparative studies of manual and algorithmic whole-breast and FGT segmentation have demonstrated superior performance of deep learning architectures, including U-Net-based models, over traditional techniques [29]. Beyond improved reproducibility, these methods enable more accurate BPE assessment and support the development of more robust predictive models.

**Table 1:** Quantitative BPE papers.

Author (Year)	Data Sources	Data Type	# of Pts	Ground Truth	Pre-Process	AUC or HR	Outcome
Arasu et al. (2020) [19]	UCSF, San Francisco, CA, USA	T0 (pre-treatment), T1 (early), T2 (during regimen), T3 (pre-surgery) Prospective	29 pCR 59 non-pCR HER2− stage II or III	Difference in pCR rates	BPE measured via fuzzy-clustering of contralateral breast	Highest cross-validated AUC of 0.81 (95% CI: 0.73–0.90) with combined FTV (pCR prediction value) + HR predictors, adding BPE to FTV + HR models had estimated AUC of 0.82	pCR
Li et al. (2020) [24]	UCSF, San Francisco, CA, USA	Baseline (T0), early (T1), inter-regimen (T2), pre-surgery (T3)	60 HR+/HER2+,162 HR+/HER2−, 30 HR−/HER2+, 132 HR−/HER2− (TNBC) from I-SPY	pCR after surgery	BPE calculated using automated fuzzy-clustering tissue segmentation	Double positive had highest AUC at 0.76 with just BPE	pCR
Nguyen AA et al. (2020) [23]	UCSF, San Francisco, CA, USA	Dynamic contrast-enhanced MRI (DCE-MRI) at baseline (T0), after 3 weeks (T1), after 12 weeks (T2), and after completion of treatment before surgery (T3)	HR+/HER2+: 57 HR+/HER2−: 140 HR−/HER2+: 27 HR−/HER2−: 116 I-SPY data	pCR after surgery	Three bilateral subvolumes analyzed: full, half, center, continuous variable (Mean early enhancement predicted pCR using AUC)	Statistically significant and highest AUC (0.87) in HR−/HER2+ at early timepoint (T1), early half-stack; vs. early full-stack AUC = 0.78 and early center 5 AUC = 0.78	pCR
Moliere et al. (2019) [25]	Department of Women’s Imaging, Strasbourg University Hospital, Strasbourg, France	Pre- and post-NAC	84 received Epirubicine + 5-Fluoro-uracile + Cyclophosphamide, 51 received additional weekly treatment with taxane and 33 patients received Trastuzumab therapy	Recurrence-free survival (local, regional, or distant); median follow-up 37 months	Semi-automated segmentation, threshold based (Used: 20%, higher post quantitative BPE (measured after NAC, before surgery) predicted recurrence on multivariable Cox)	HR = 6.38, *p* < 0.05 for post-NAC BPE predicting recurrence	RFS, pCR
Rella R et al. (2020) [26]	Fondazione Policlinico Universitario A. Gemelli IRCCS, Rome, Italy	Post-NAC	228 patients with breast cancer	ROI analysis of enhancement kinetics performed by two radiologists in consensus	Semi-automated contralateral ROI segmentation assessed enhancement change	N/A	pCR
Onishi et al. (2021) [20]	UCSF, San Francisco, CA, USA	Baseline (T0), early (T1), inter-regimen (T2), pre-surgery (T3)	882 from I-SPY (hormone receptor + and—cohorts) HR positive and HR-negative groups analyzed separately	Difference in pCR rates	Fully automated segmentation; central 50% of axial sections of contralateral breast as target volume; fuzzy c-means clustering algorithm for segmentation of FGT; voxel-by-voxel early percent enhancement map (non-contrast and early contrast-enhanced phase) computed as follows: [(SI post – SI pre/SI pre] × 100%. Averaged percent enhancement values for all voxels in masked volume to generate quantitative BPE.	No AUC given; lack of BPE suppression and lower pCR rate in the HR-positive cohort at T2 (*p* = 0.02) and T3 (*p* = 0.003)	pCR
Rella R. et al. (2022) [28]	Fondazione Policlinico Universitario A. Gemelli IRCCS, University Cattolica Sacro Cuore, Rome, Italy.	Baseline MRI was performed within four weeks before NAC‚ post-NAC MRI within two weeks after completion of chemotherapy.	30 pCR, 198 non-pCR	pCR defined as absence of residual invasive cancer cells in the breast and ipsilateral lymph nodes	FGT manually segmented, BPE rate calculated as [(SIpostCM − SIpreCM)/SIpreCM] × 100%.	Higher stage (HR 3.6), Ki-67 (1.9), ypN (2.0) worsen survival	OS, DFS
You et al. (2017) [21]	Fudan University Cancer Center, Shanghai, China	Baseline (T0), after 2nd, 4th, 6th NAC	90 unilateral breast cancer 25 pCR/65 non-pCR	pCR after surgery	Fully auto whole breast segmentation → FGT segmentation → enhanced FGT segmentation	AUC = 0.726 for ΔBPE predicting pCR highest at early timepoint (after 2nd NAC) Larger magnitude for change in BPE in HR-negative group	pCR
Chen et al. (2015) [22]	University of California, Irvine, USA	Pre-treatment MRI, 2 follow-up MRIs during ongoing NAC	46 total 24 pCR/22 non-pCR	pCR on surgical pathology	Auto averaged enhanced FGT segmentation	AUC N/A Higher pre-treatment BPE if pCR. Compared to baseline, BPE at F/U-1 significantly decreased in pCR. Subgroup analysis by age: only seen in the younger group (<55 years old), not in the older group (≥55 years old). Older patients significantly lower pre-treatment BPE. Significantly decreased BPE at F/U-1 only in the ER-negative pCR group but not non-pCR, or ER-positive groups.	pCR
Shin G et al. (2019) [27]	Yonsei University College of Medicine, Seoul, Korea	Preoperative DCE-MRI (contralateral breast)	289 unilateral ER+/HER2−, node-negative invasive breast cancer (>5 mm)	RFS and distant metastasis-free survival	Quantitative BPE (manual ROI-based as well as fully automated segmentation) in addition to qualitative BPE assessment by two radiologists using BI-RADS	AUC N/A Contralateral BPE not associated with RFS or DFS (*p* > 0.05). Ki-67 expression level was associated with worse survival outcome.	RFS, DFS
van der Velden et al. (2018) [14]	Memorial Sloan Kettering Cancer Center, USA	Pre-treatment DCE-MRI (contralateral parenchyma)	Biomarker-assessment study (ER+/HER2− invasive ductal carcinoma) (*n* = 302)	Invasive DFS (IDFS), OS ER+/HER2−	Auto contralateral parenchyma segmentation → late-phase enhancement computations	Pre-treatment BPE independent biomarker for Survival: IDFS HR = 0.27, OS HR = 0.22	IDFS, OS

## 4. Conclusions

The quantitative assessment of BPE is a more objective and reproducible approach than the qualitative methods, which often are subjective and are prone to inter-reader variability. Quantitative BPE eliminates subjectivity and reduces observer bias and variability between studies by providing a numerical measurement approach. However, the reliability of quantitative measurements is dependent on strict methodology. This includes standardization of segmentation techniques, correct timing of imaging, and overall consistent acquisition parameters for data. Without these strict criteria for methodology, the results of quantitative measurements may vary widely, which limits generalizability and interpretability. Virtually all of the quantitative studies that utilized automated segmentation found some association between BPE and prognosis/pCR. Further refinements, such as utilization of a percent threshold for inclusion in BPE calculation as employed by Moliere et al., may strengthen these analyses.

The literature increasingly supports fully automated approaches for quantifying BPE across multiple timepoints, as these methods are more reliable and reproducible than qualitative or semi-automated assessments. Manual intervention in ROI segmentation can introduce variability and reduce measurement accuracy, underscoring the need for automated solutions.

Given the critical role of accurate FGT segmentation and the absence of a standardized methodology, deep learning-based approaches offer a promising alternative. This also provides an opportunity to integrate BPE data with broader clinical and imaging data. Such a combination allows for even greater analysis than with BPE data alone. In a clinical setting, these advancements could improve prediction and treatment monitoring in oncology. Integration of this imaging biomarker with molecular or histologic tumor features, patient risk factors, genetics (e.g., BRCA status), hormone receptor status, and other relevant variables may prove to be very informative. Inclusion of quantitative BPE in machine learning prediction models may allow more accurate prediction of outcomes and may help in guiding treatment. A current limitation is that many of the quantitative BPE studies are retrospective and based on modest sample sizes, which limits generalizability. There is a clear need for larger, prospective, multi-institutional cohorts employing harmonized imaging acquisition parameters, standardized definitions of quantitative BPE metrics, and consistent post-processing methods. Large-scale validation using standardized protocols will be essential before quantitative BPE can be reliably integrated into routine clinical practice.

## Data Availability

Data is available on PubMed. See “Section 2” for our process.

## Abbreviations

The following abbreviations are used in this manuscript:

BPE	Background parenchymal enhancement
MRI	Magnetic resonance imaging
pCR	Pathologic complete response
RFS	Recurrence-free survival
DFS	Disease-free survival
OS	Overall survival
NAC	Neoadjuvant chemotherapy
BI-RADS	Breast imaging-reporting assessment and data system
HR	Hormone receptor
HER2	Human epidermal growth factor receptor
FTV	Functional tumor volume
FGT	Fibroglandular tissue
AUC	Area under the receiver operating characteristic curve
ROI	Region of interest
BRCA	Breast cancer gene
TNBC	Triple-negative breast cancer
DCE-MRI	Dynamic contrast-enhanced MRI
IDFS	Invasive DFS
